# Post-COVID-19 fatigue and health-related quality of life in Saudi Arabia: a population-based study

**DOI:** 10.3389/fpubh.2023.1254723

**Published:** 2023-10-05

**Authors:** Moath S. Al-Johani, Rehana Khalil, Yazeed A. Al-Mohaimeed, Omar M. Al-Mundarij, Abdulmajeed S. Al-Samani, Osama S. Al-saqry, Alwaleed A. Al-saawi, Ibrahim K. Al-dhali, Waleed A. Al-Essa

**Affiliations:** ^1^Department of Family and Community Medicine, Unaizah College of Medicine and Medical Sciences, Qassim University, Unaizah, Saudi Arabia; ^2^Research Unit, Unaizah College of Medicine and Medical Sciences, Qassim University, Unaizah, Saudi Arabia

**Keywords:** post-COVID-19, fatigue, quality of life, SF12, CFQ 11, Saudi Arabia, public health, epidemiology

## Abstract

**Background:**

Despite substantial literature on symptoms and long-term health implications associated with COVID-19; prevalence and determinants of post-acute COVID-19 fatigue (PCF) remain largely elusive and understudied, with scant research documenting health-related quality of life (HRQoL). Hence, prevalence of PCF and its associated factors, and HRQoL among those who have survived Covid-19 within the general population of Saudi Arabia (KSA) is the subject under examination in this research.

**Methods:**

This cross-sectional study was conducted on 2063 individuals, selected from the KSA’s general population, using a non-probability sampling approach. An online survey was used to employ a self-administered questionnaire to the participants, which included socio-demographic information, the patient’s COVID-19 infection history, 12-item Short Form Health Survey (SF-12) to assess quality of life, and Chalder Fatigue Scale (CFS) (CFQ 11) to evaluate the extent and severity of fatigue. Data were analyzed using SPSS version 26. A *p* < 0.05 was considered to be strong evidence against the null hypothesis.

**Results:**

The median age of participants was 34 (IQR = 22) years, with females comprising the majority (66.2%). According to the SF-12 questionnaire, 91.2% of patients experienced physical conditions, and 77% experienced depression. The prevalence of PCF was 52% on CFQ 11 scale. Female gender, higher levels of education, a pre-existing history of chronic disease, as well as the manifestations of shortness of breath and confusion during acute COVID-19 infection, were identified as independent predictors of fatigue.

**Conclusion:**

To facilitate timely and effective intervention for post-acute COVID-19 fatigue, it is essential to continuously monitor the individuals who have recovered from acute COVID-19 infection. Also, it is critical to raise health-education among these patients to improve their quality of life. Future research is required to determine whether COVID-19 survivors would experience fatigue for an extended duration and the impact of existing interventions on its prevalence and severity.

## 1. Introduction

Over the past two decades, there have been two coronavirus outbreaks, namely severe acute respiratory syndrome coronavirus 1 (SARS-CoV-1) and Middle East respiratory syndrome coronavirus (MERS-CoV) (1). Both of these viruses have been linked with enduring post-disease effects in numerous individuals (2, 3). A new species (SARS-CoV-2) of this family emerged in December of 2019, leading to a global COVID-19 pandemic (4). Given that the COVID-19 virus is closely related to MERS and SARS, it seems reasonable to anticipate a higher probability of post-disease manifestations (5). The comprehensive range of clinical symptoms that occur following acute phase of COVID-19 infection has been labeled as “post-COVID-19 syndrome (PCS)” by the National Institute for Health and Care Excellence (NICE). PCS is characterized by new and/or persistent clinical manifestations that persist for a duration of more than 12 weeks following the initial acute infection (6).

Based on existing research on COVID-19, common post-acute symptoms encompass diarrhea, depression, arthralgia, headache, vertigo, sleep disturbances, post-exertional dyspnea, chronic cough, and fatigue (7, 8). A considerable amount of medical literature has been examined in a systematic review and meta-analysis which showed that PCS patients exhibited symptoms predominantly related to the musculoskeletal, nervous system, and digestive system (9). Arthralgia and diarrhea were experienced by more than 40% of PCS patients. Moreover, a recent study revealed that 30% of the individuals had at least one post-COVID-19 symptom, and 16% had multiple symptoms (10). Post-COVID-19 fatigue (PCF) was found to be the most prevalent, persistent, and primary complaint in several contemporary studies (11–16). Evidence suggests that PCF not just has an impact on physical, or mental health, but on overall quality of life (17).

According to a research conducted in Egypt, the presence of fatigue, cognitive impairment, stress, depression, sleep difficulties, and frequent falls in the recovery phase of COVID-19 were all found to be strongly associated with PCF (18). The prevalence of PCF in post-hospitalized patients varies between 52 and 70% at 1–3 months after discharge from the hospital (8, 19, 20). While the non-hospitalized COVID-19 patients represent a comparatively large proportion of population than hospitalized, but most of the present-day information is focused on hospitalized ones (21). In a sample of non-hospitalized individuals, a proportion of 50-75% reported an experiencing fatigue during the COVID-19 pandemic, but there is a paucity of information with regards to the long-term follow-up (22). Another study conducted in the general population indicated 24% ongoing fatigue during follow-up ensured via telephone (23). The aforementioned studies employed single item to assess fatigue (22, 23). There is a dearth of information regarding the use of more exhaustive questionnaires designed to assess fatigue, except one recent study which demonstrated that fatigue was present in 52% of their sample on Chalder fatigue scale (CFQ-11) (24).

Upon conducting a comprehensive examination of the extant literature, it has been discerned that several investigations have been done worldwide on the persistence of symptoms post-acute COVID-19 infection. However, the prevalence of post-acute COVID fatigue (PCF) has only been examined in limited studies, with the majority of inquiries relying on general questionnaires rather than specific ones. Moreover, research work on the persistent symptoms following recovery from acute COVID-19 in Saudi Arabia is scarce (25–27). Additionally, there have been no inquiries into PCF and HRQoL. Our research aimed to evaluate the post-COVID-19 fatigue (PCF) utilizing the CFQ-11 questionnaire, and to identify potential risk factors that contribute to persistent fatigue. Furthermore, the study sought to determine the health related quality of life (HRQoL) in COVID-19 Survivors. A comprehensive understanding of the HRQoL, and relationship between PCF and its predictors, would enable medical practitioners to be more precise in choosing appropriate strategies to optimize care of post-COVID-19 patients, and deal with any future coronavirus outbreak in the Kingdom of Saudi Arabia.

## 2. Materials and methods

### 2.1. Study design, participants, and procedure

This cross-sectional study was carried out from June to September 2022, among the general population of the KSA using a non-probability sampling technique. An online survey (SurveyMonkey) was used to send a self-administered questionnaire to the participants, and the study link was distributed using social media networks such as Twitter and WhatsApp. The inclusion criteria were (i) COVID-19 infected subjects, (ii) age ≥ 18 years, (iii) inhabitants of KSA, and (iv) willing to participate in the study.

### 2.2. Data collection tool

The questionnaire was developed on the basis of recently published literature to address the study’s objectives (21, 24, 28–32). A pilot study using Arabic version of the questionnaire was conducted on 35 subjects and data were not included in the final results. The final Arabic version of the questionnaire was reviewed by experts to ensure face and content validity.

The objective of the study along with the inclusion/exclusion criteria, the time required for survey completion, and the contact information of the study investigators were mentioned on the cover page of the survey, and consent to participate was needed before filling out the questionnaire. The questionnaire comprised of four sections. Section 1 was about socio-demographic factors (age, gender, marital status, level of education, smoking status, BMI, and history of chronic disease). Section 2 included COVID-19 infection history, COVID-19 vaccine, post-acute COVID-19 symptoms, hospitalization, and time elapsed since diagnosed with COVID-19 infection. Section 3 involved SF-12 HRQoL assessment and Section 4 consisted of the Chalder Fatigue Scale (CFS) (CFQ-11).

#### 2.2.1. Fatigue assessment

The PCF was assessed using the self-administered validated Arabic version of Chalder Fatigue Scale (CFQ-11), which is used to assess fatigue symptoms in clinical and non-clinical settings (31, 33, 34). It has 11 items on an ordinal scale of 0–3, which are summed together to get a total score range from 0 to 33 and it also covers physical (0–21) and psychological (0–12) fatigue domains (28). Moreover, CFQ-11 also provides fatigued vs. non-fatigued case-status (a cut-off at <4 vs. ≥4) based on bimodal scoring which dichotomizes the response of 0 and 1 (“Better than usual”/“No worse than usual”) as zero score and 2 and 3 (“Worse than usual”/“Much worse than usual”) as 1 score (28, 34). We utilized case-status (fatigue vs. non-fatigued) using the bimodal scoring method for the current study with a cut-off at <4 vs. ≥4 (21, 24, 35).

#### 2.2.2. HRQoL assessment

The health-related quality of life (HRQoL) of the participants was assessed using validated Arabic version of SF-12 which comprises of same eight domains of SF-36 (29, 32, 36). The SF-12 questionnaire has 12 items and two subscales: the physical health component scale (PCS) and the mental health component scale (MCS). Each subscale comprises four domains: PCS: physical functioning, role physical, bodily pain, and overall health; MCS: vitality, social functioning, role emotional, and mental health. The PCS and MCS were classified based on their overall score (29, 30). A score of 50 or less on the PCS-12 has been recommended as a cut-off for determining a physical condition, but a score of 42 or less on the MCS-12 may indicate “clinical depression” (30).

### 2.3. Sample size calculation

The minimum sample size was determined with Raosoft software, and it was estimated to be 490. The assumptions included a response distribution of 50% to attain the maximum sample size, a 5% margin of error, a confidence interval (CI) of 95, and 30% added to account for incomplete or missing responses.

### 2.4. Statistical analysis

The data were analyzed using SPSS version 26 (Armonk, NY: IBM Corp, United States). Numbers, percentages, the mean, and standard deviation (SD) were used to summarize descriptive statistics. The Chi-square test examined the association between fatigue levels, and physical and mental health conditions in relation to predictors. Significant findings were then incorporated into multivariate regression models to establish the significant independent predictors of fatigue, along with the corresponding adjusted odds ratio and 95% confidence range. A *p* < 0.05 was considered to be strong evidence against the null hypothesis.

## 3. Results

A total of 2063 participants were included in the study. The baseline characteristics of the participants are presented in Table 1. The participants’ median age was 34 (IQR = 22) years, with 51.6% of them being of age below 35 years. Females (66.2%) outnumbered males (33.8%). Just over half of the participants (56.6%) were married, besides 59.9% had a bachelor & higher education degree. Most of them (86.5%) were non-smokers, while 59% were overweight or obese, and 55.5% had a history of chronic disease. Among all subjects majority were infected only once (85.1%) and rest had COVID-19 infection more than once. A duration of 10 months or less was elapsed for half (50.8%) of the participants since their diagnosis of COVID-19 infection. The proportions of participants with shortness of breath and confusion were 46.5 and 36.2%, respectively. Only 2.8% of the participants were admitted to the hospital for COVID-19 infection. Almost three-quarter (72.9%) of the participants had a history of vaccination against COVID-19 before contracting COVID-19 infection.

**Table 1:** Sociodemographic, general health, and COVID-19 related data of the study participants (*n* = 2063).

Variable	N (%)
Age group [median (IQR)]	34 (22)
<35 years	1064 (51.6%)
≥35 years	999 (48.4%)
Gender	
Male	698 (33.8%)
Female	1365 (66.2%)
Marital status	
Unmarried	896 (43.4%)
Married	1167 (56.6%)
Level of education	
Diploma and below	828 (40.1%)
Bachelor and higher education	1235 (59.9%)
Smoking status	
Current or former smoker	279 (13.5%)
Non-smoker	1784 (86.5%)
BMI level	
Normal (18.5–24.9 kg/m^2^) or Underweight (<18.5 kg/m^2^)	846 (41.0%)
Overweight (25–29.9 kg/m^2^) or Obese (≥30 kg/m^2^)	1217 (59.0%)
History of chronic disease	
No	919 (44.5%)
Yes	1144 (55.5%)
History of COVID-19 infection	
Yes, at least once	1765 (85.1%)
Yes, more than once	307 (14.9%)
Having shortness of breath during COVID-19 infection	
No	1104 (53.5%)
Yes	959 (46.5%)
Experienced confusion during COVID-19 infection	
No	1315 (63.8%)
Yes	745 (36.2%)
Time since diagnosed with COVID-19 infection	
10 months or less	1048 (50.8%)
More than 10 months	1015 (49.2%)
History of vaccination prior COVID-19 infection	
No	595 (27.1%)
Yes	1504 (72.9%)
Hospitalization due to COVID-19	
No	2006 (97.2%)
Yes	57 (2.8%)

The SF-12 and CFS (CFQ-11) descriptive statistics are displayed in Table 2. The mean physical component score (PCS) on the SF-12 was 42.4 (SD 6.36). The PCS domains’ mean scores for physical functioning, role physical, bodily discomfort, and general health were 4.76, 0.85, 4.06, and 4.03, respectively. Based on the overall score, 91.2% of those who took the test were classified as having a physical condition, and the remaining 8.8% were normal. The mean mental component score (MCS) was 37.6 (SD 6.13). The MCS domains’ respective mean scores for vitality, social functioning, emotional role, and mental health were 3.94, 2.44, 0.89, and 8.26. Over three-quarters (77%) were found to have depression, while 23% did not. The overall mean fatigue score on CFS (CFQ-11) was 4.17 (SD 3.58). Around half (52%) of the of the participants were categorized as fatigued, while 48% were normal.

**Table 2:** Descriptive statistics of SF-12 form and Chalder fatigue scale (CFQ-11) (*n* = 2,063).

SF-12 domains	N (%)
Total physical component score (mean ± SD)	42.4 ± 6.36
Physical functioning score	4.76 ± 1.23
Role physical score	0.85 ± 0.89
Bodily pain score	4.06 ± 0.95
General health score	4.03 ± 0.99
PCS level	
With physical condition (score ≤50)	1881 (91.2%)
Without physical condition (score >50)	182 (8.8%)
Total mental component score (mean ± SD)	37.6 ± 6.13
Vitality score	3.94 ± 1.18
Social functioning score	2.44 ± 1.25
Role emotional score	0.89 ± 0.92
Mental health score	8.26 ± 2.21
MCS level	
With depression (score ≤42)	1588 (77.0%)
Without depression (score >42)	475 (23.0%)
Total fatigue score (mean ± SD)	4.17 ± 3.58
Level of fatigue	
Fatigue (score ≥4)	1072 (52.0%)
Normal (score <4)	991 (48.0%)

Table 3 shows that the study found that post-COVID-19 fatigue (PCS) was more common among females (*p* < 0.001), those with better education (*p* = 0.029), non-smokers (*p* = 0.010), overweight and obese (*p* = 0.027), those without chronic diseases (*p* < 0.001), those experiencing shortness of breath infection (*p* < 0.001), confusion (*p* < 0.001), those diagnosed for 10 months or less (*p* < 0.001), and those who received vaccinations before the infection (*p* = 0.003). Physical conditions were significantly related with the participants who were of older age group (*p* = 0.019), married (*p* = 0.005), overweight and obese (*p* = 0.023), and those without fatigue (*p* = 0.004). Participants with depression were more likely to be older adult (*p* = 0.036), married (*p* = 0.012), and fatigued (*p* = 0.007).

**Table 3:** Relationship between the level of fatigue, physical health and depression with socio-demographic, and COVID-19 infection related factors of the participants (*n* = 2,063).

	Level of fatigue		*P*-value^§^	Level of physical health		*P*-value^§^	Level of depression		*P*-value^§^
	Fatigue N (%) (*n* = 1072)	Normal N (%) (*n* = 991)		Physical condition N (%) (*n* = 1881)	No physical condition N (%) (*n* = 182)		Depressed N (%) (*n* = 1588)	Not depressed N (%) (*n* = 475)	
Age group (mean ± SD)									
<35 years	574 (53.5)	490 (49.4)	0.063	955 (50.8)	109 (59.9)	0.019**	799 (50.3)	265 (55.8)	0.036**
≥35 years	498 (46.5)	501 (50.6)		926 (49.2)	73 (40.1)		789 (49.7)	210 (44.2)	
Gender									
Male	258 (24.1)	440 (44.4)	<0.001**	642 (34.1)	56 (30.8)	0.360	526 (33.1)	172 (36.2)	0.212
Female	814 (75.9)	551 (55.6)		1239 (65.9)	126 (69.2)		1062 (66.9)	303 (63.8)	
Marital status									
Unmarried	474 (44.2)	422 (42.6)	0.455	799 (42.5)	97 (53.3)	0.005**	666 (41.9%)	230 (48.4%)	0.012**
Married	598 (55.8)	569 (57.4)		1082 (57.5)	85 (46.7)		922 (58.1%)	245 (51.6%)	
Level of education									
Diploma and below	406 (37.9)	422 (42.6)	0.029**	763 (40.6)	65 (35.7)	0.203	630 (39.7)	198 (41.7)	0.433
Bachelor and higher education	666 (62.1)	569 (57.4)		1118 (59.4)	117 (64.3)		958 (60.3)	277 (58.3)	
Smoking status									
Current or former smoker	125 (11.7)	154 (15.5)	0.010**	253 (13.5)	26 (14.3)	0.753	210 (13.2)	69 (14.5)	0.467
Non-smoker	947 (88.3)	837 (84.5)		1628 (86.5)	156 (85.7)		1378 (86.8)	412 (85.5)	
BMI									
Normal (18.5–24.9 kg/m^2^) or Underweight (<18.5 kg/m^2^)	415 (38.7)	431 (43.5)	0.027	757 (40.2)	89 (48.9)	0.023**	644 (40.6)	202 (42.5)	0.443
Overweight (25–29.9 kg/m^2^) or Obese (≥30 kg/m^2^)	657 (61.3)	560 (56.5)		1124 (59.8)	93 (51.1)		944 (59.4)	273 (57.5)	
History of chronic disease									
No	604 (56.3)	315 (31.8)	<0.001**	840 (44.7)	79 (43.4)	0.746	726 (45.7)	193 (40.6)	0.050
Yes	468 (43.7)	676 (68.2)		1041 (55.3)	103 (56.6)		862 (54.3)	282 (59.4)	
Having shortness of breath during COVID-19 infection									
No	503 (46.9)	601 (60.6)	<0.001**	1005 (53.4)	99 (54.4)	0.803	853 (53.7)	251 (52.8)	0.738
Yes	569 (53.1)	390 (39.4)		876 (46.6)	83 (45.6)		735 (46.3)	224 (47.2)	
Experienced confusion during COVID-19 infection									
No	541 (50.5)	774 (78.2)	<0.001**	1202 (63.9)	113 (62.1)	0.620	1010 (63.6)	305 (64.3)	0.767
Yes	531 (49.5)	216 (21.8)		678 (36.1)	69 (37.9)		578 (36.4)	169 (35.7)	
Time since diagnosed with COVID-19 infection									
10 months or less	596 (55.6)	452 (45.6)	<0.001**	960 (51.0)	88 (48.4)	0.489	809 (50.9)	239 (50.3)	0.810
More than 10 months	476 (44.4)	539 (54.4)		921 (49.0)	94 (51.6)		779 (49.1)	236 (49.7)	
History of vaccination prior COVID-19 infection									
No	261 (24.3)	298 (30.1)	0.003**	512 (27.2)	47 (25.8)	0.686	430 (27.1)	129 (27.2)	0.973
Yes	811 (75.7)	693 (69.9)		1369 (72.8)	135 (74.2)		1158 (72.9)	346 (72.8)	
Hospitalization due to COVID-19									
No	1037 (96.7)	969 (97.8)	0.148	1826 (97.1)	180 (98.9)	0.151	1549 (97.5)	457 (96.2)	0.120
Yes	35 (03.3)	22 (02.2)		55 (02.9)	02 (01.1)		39 (02.5)	18 (03.8)	
Level of fatigue									
Fatigue	–	–	–	959 (51.0)	113 (62.1)	0.004**	851 (53.6)	221 (46.5)	0.007**
Normal	–	–		922 (49.0)	69 (37.9)		737 (46.4)	254 (53.5)	

^§^*P*-value has been calculated using Chi-square test. **Significant at *p* < 0.05 level.

In multivariate regression model displayed as Table 4, females were predicted to have a 1.9 times higher risk of fatigue (AOR 1.945, 95% CI 1.558–2.428, *p* < 0.001) as compared to male participants of the study. Those with an education level of bachelor or higher had a 1.26-fold higher risk of fatigue than those with a lower level (Diploma or below) of education (AOR 1.262, 95% CI 1.040–1.532, *p* = 0.018). Participants with shortness of breath during COVID-19 infection were 1.29 times (AOR 1.297, 95% CI 1.067–1.577, *p* = 0.009). and with confusion during COVID-19 infection were 3 times (AOR 3.032, 95% CI 2.466–3.728, *p* < 0.001) more likely to suffer from fatigue. On the other hand, participants with a history of chronic disease, were predicted to have a lower risk of fatigue (AOR 0.414, 95% CI 0.341–0.504, *p* < 0.001) as compared to those without any history of chronic disease. Also, the patients with more than 10 months elapsed since their diagnosis of COVID-19 infection were predicted to have a lower risk of fatigue (AOR 0.662, 95% CI 0.535–0.819, *p* < 0.001) than those with less than 10 months of COVID-19 diagnosis.

**Table 4:** Multivariate regression analysis to determine the independent significant predictors of post-COVID-19 fatigue (PCF) (*n* = 2063).

Variable	COR (95% CI)	*P*-value	AOR	95% CI	*P*-value
Gender					
Male	Ref		Ref		
Female	2.519 (2.088–3.040)	0.001	1.945	1.558–2.428	<0.001**
Level of education					
Diploma or below			Ref		
Bachelor or higher	1.217 (1.020–1.451)	0.029	1.262	1.040–1.532	0.018**
Smoking status					
Current or former smoker	Ref		Ref		
Non-smoker	1.394 (1.082–1.796)	0.010	0.922	0.681–1.248	0.599
BMI					
Normal (18.5–24.9 kg/m^2^) or Underweight (<18.5 kg/m^2^)	Ref		Ref		
Overweight (25–29.9 kg/m^2^) or Obese (≥30 kg/m^2^)	1.218 (1.022–1.453)	0.028	1.064	0.876–1.293	0.531
History of chronic disease					
No	Ref		Ref		
Yes	0.361 (0.302–0.432)	0.001	0.414	0.341–0.504	<0.001**
Shortness of breath during COVID19 infection					
No	Ref		Ref		
Yes	1.743 (1.463–2.077)	0.001	1.297	1.067–1.577	0.009**
Experienced confusion during COVID19 infection					
No	Ref		Ref		
Yes	3.517 (2.901–4.264)	0.001	3.032	2.466–3.728	<0.001**
Time since diagnosed with COVID19 infection					
10 months or less			Ref		
More than 10 months	0.670 (0.563–0.797)	0.001	0.662	0.535–0.819	<0.001**
History of vaccination prior to COVID19 infection					
No	Ref		Ref		
Yes	1.363 (1.100–1.623)	0.004	1.082	0.851–1.376	0.520

AOR, Adjusted odds ratio; CI, Confidence Interval. **Significant at *p* < 0.05 level.

## 4. Discussion

Our results demonstrate that the prevalence of fatigue among COVID-19 survivors was 52%, which is much higher than the prevalence (11.5%) reported by previous Saudi study (27). A plausible rationale behind this disagreement could be the difference in assessment methods and temporal proximity to the onset of acute COVID-19 infection between two studies. The aforementioned Saudi study employed a general identification questionnaire for post-acute COVID-19 symptoms, unlike the present study’s utilization of a specialized fatigue measurement scale, CQF-11. Furthermore, the former study reported symptoms surpassing 4 weeks following the diagnosis of acute COVID-19 infection, while our data documented fatigue based on criteria of 10 months or more. A surprisingly similar prevalence of 52% was found by an Irish study, which used the CQF-11 and same cut-off to define fatigue as in the current study (24). Our finding is also comparable with a recent study conducted in Egypt, which reported that the estimated prevalence of chronic fatigue syndrome (CFS) was 60%, supported by another contemporary Sweden based study that found a fatigue prevalence of 64.4% (18, 37). Nevertheless, a Chinese study based on multi-item scale, declared a prevalence of 53% after 4 weeks of hospitalization for COVID-19 infection (38). Also, a study in Netherlands reported 69% of fatigue on a clinical screening instrument after 3 months of hospitalization for COVID-19 infection (39). Even though, it would not be a misapprehension that the prevalence of fatigue appears to be higher than 50% in multiple studies conducted worldwide, but the differences in methods, populations, and timing relative to the acute COVID-19 infection phase could complicate the comparisons across studies. For example, some studies employed single item tools to assess post COVID-19 fatigue (8, 19, 20, 40). Moreover, about 60–70% fatigue was reported among hospitalized patients 48 days after their hospital discharge by one study, while another research revealed 53% of hospitalized patients on average 36 days after hospital discharge, compared to the study which showed 87% fatigue on average 79 days after onset of COVID-19 infection (8, 19, 40). Notwithstanding the uncertain evidence about temporal connection, our results support the notion that participants with more than 10 months elapsed since their diagnosis of COVID-19 infection were predicted to experience a decrease in the likelihood of experiencing fatigue.

In the current study, being female was found to be associated with fatigue. This finding is inconformity with other contemporary studies conducted on the subject of persistent fatigue in post-acute phase of COVID-19 (21, 24, 41–43). Previous research aimed at comprehending fatigue in general population suggests that a greater level of fatigue is experienced by females in specific situations as compared to males (35, 44, 45). However, the existing evidence is not conclusive (46).

We found a higher likelihood of post-acute COVID-19 fatigue among participants with a higher educational level. Inconsistent findings have emerged in previous studies on the relationship between education and fatigue as a long-term symptom of COVID-19 infection. For instance, a nationwide registry-based study carried out in Sweden found no correlation between education and post-acute COVID-19 fatigue (47). Conversely, other study demonstrated that individuals with lower levels of education are at a higher risk of developing persistent symptoms following an acute COVID-19 infection (48).

The results of the present study indicate that the history of chronic disease does not appear to have a significant impact on the likelihood of experiencing fatigue. However, there is evidence that complaint of fatigue is very common among people with chronic illnesses (49). This could potentially be explained by various rationales. One of the possible explanations could be a difficulty of those participants to quantify any change in their existing chronic fatigue which they used to bear due to their preexisting chronic illness. Another conceivable explanation is that our study subjects did not have any chronic illnesses that typically yield fatigue. Alternatively, it could be posited that fatigue may take a longer time to develop in case of chronic conditions and was not observable in our sample. Moreover, the age range of our participants makes it plausible that any chronic diseases they have not yet caused fatigue symptoms. Consequently, it may be inferred that individuals who lack certain factors related to chronic medical conditions either before, during or after acute COVID-19 infection may have a reduced susceptibility to post-infection fatigue. This necessitates further exploration to assess the potential association between the risk of post-COVID-19 fatigue and a history of chronic disease.

Other predictors of fatigue in our study during the post-acute phase included shortness of breath and confusion during acute COVID-19. These findings are in line with an existing study showing that fatigue is linked to shortness of breath and confusion during acute phase of COVID-19 (21).

Our findings on respondents’ perception of their own health status showed that a vast majority or nearly all (91.2%) had a score of 50 or less on the PCS-12 indicating they had a physical condition, while 77% scored less than 42 on the MCS-12 indicating having a clinical depression that interfered with their everyday activities. Different findings from the studies on the association between COVID-19 severity in post-acute symptoms development and declining HRQoL have been reported, which may be due to variations in the methodologies and samples used. A study by Halpin et al., found a relationship between COVID-19 severity and poorer HRQoL, whereas Garrigues et al. found no differences between patients in the ICU and Wards in terms of their frequency of post-acute symptoms development and decreased health-related quality of life (HRQoL) on the basis of their acute COVID-19 severity (19, 50). Nonetheless, other research studies have concluded that patients who experience post-COVID-19 syndrome (PCS) are likely to report a lower HRQoL (8, 19, 51, 52). The most prevalent physical symptoms related to post-acute COVID-19 reported in literature include fatigue, anosmia, dyspnea, cough, insomnia, arthralgia, palpitations, chest pain, and headache (27, 53–58). Even though the mechanism behind post-acute physical symptoms still unclear but, Doykov et al. reported that inflammatory markers and upregulation of mitochondrial protein in COVID-19 patients lead to mitochondrial stress 40–60 days after infection (59). This can cause cytokine dysregulation and eventually to the cytokine release storms. Additionally, it is evident through autopsies that Coronavirus can spread to the central nervous system through nerves. So, the cytokine storm along with the virus entry to CNS can result in neuroinflammation and produce symptoms like fatigue, myalgias, headache, dyspnea, and psychiatric consequences (60–62).

The potential long-term cause of poor mental quality of life due to PCS is linked to anxiety, cognitive impairments, and depressive symptoms (63, 64). Factors including social isolation, financial costs, and prolonged symptoms have a negative impact on mental health and their perceived quality of life (65–67). At 1-, 3-, 6-, and 12-month follow-up after being infected with SARS-CoV-2, about 30–40% of patients were found with clinically significant depressive psychopathology (68–72). Depressive symptoms, including depressed mood, cognitive impairment, and diminished interest, seem to have a negative impact on everyday quality of life. Both pre-existing history of depression and COVID-19-related depressive symptoms were identified as risk factors for poor SARS-CoV-2 infection outcomes such as higher infection rate, hospitalization, admission in intensive care unit, and mortality (73, 74). We found a high rate of depression in subjects on SF-12 and this could explain high prevalence of fatigue on chalder fatigue scale. The notion of association between depression after COVID-19 with Post-COVID-19 fatigue (PCF) is supported by several studies (24, 75, 76). It appears that there is a shared pathophysiological mechanism between post-COVID fatigue and post-COVID depression, which could potentially be related to indirect immune-inflammatory mediated neuroinflammation (61, 77). Numerous studies have hypothesized and confirmed a causal relationship between systemic inflammation and both post-COVID depression and post-COVID fatigue (55, 69, 72, 78). Furthermore, since depressive symptoms have been associated with neurocognitive functioning and quality of life in COVID-19 survivors, it seems that depression is one of the most pertinent predictors of the post-COVID syndrome (79–82). Considering alarmingly high prevalence of post-COVID-19 depression, it is the need of the hour to provide follow-up services for COVID-19 survivors to monitor mental health and provide early interventions (64, 83).

Although the current study provides information on the prevalence of post-COVID-19 fatigue (PCF) and health-related quality of life (HRQoL) assessments, its cross-sectional nature provides a measurement at a certain point in time and limits information on the specific timing and its association with acute COVID-19 infection. Given that the questionnaire was distributed via social media platforms, which may prove inadequate in accurately representing the study population owing to the fact that the study did not include the illiterate, the older adult, those devoid of smartphones, and individuals with limited internet accessibility across different regions of the kingdom. Also, a constraint of this study is the lack of specificity in the factor denoting the presence or absence of chronic disease, as it does not offer details on the type, duration, and severity of the chronic condition. Furthermore, the PCF assessment was not conducted by clinical examination in a healthcare context but through a self-reported tool, and study was restricted to assess solely the presence of shortness of breath and confusion as COVID-19 infection-related symptoms. Serial cross-sectional or prospective investigations are required to determine whether PCF prevalence rate remains stable and the influence of specific therapies on its intensity and prevalence.

## 5. Conclusion

The current study has shown that the prevalence of PCF was 52% among COVID-19 survivors. Furthermore, female, highly educated individuals, who experienced shortness of breath and confusion during COVID-19 infection were more likely to exhibit fatigue symptoms than the rest of the study participants. Results suggest that both physical and mental components scores showed poor self-perceived HRQoL in post-acute phase of COVID-19. We recommend longitudinal quantitative studies to assess patients at multiple time points, examine their immune markers, and describe fatigue persistence at every 6 months and beyond. Even though, Chalder Fatigue Scale (CFQ-11) is appropriate, but further a large cohort study is needed to identify subgroups and related complex factors. We also recommend qualitative studies to understand the subjective differences in experiences of people in their post-acute phase of this viral infection. To implement timely and efficient intervention, continuous monitoring of COVID-19 survivors should be done, and analysis of multi-disciplinary fatigue management strategies is recommended in post-COVID phase. It is critical to raise health-education among COVID-19 survivors to improve their quality of life. Participation in physical activities and mental therapies are the most significant things that our healthcare authorities can teach to COVID-19 survivors in order to improve their capacity and quality of life.

## Data availability statement

The raw data supporting the conclusions of this article will be made available by the authors, without undue reservation.

## Ethics statement

The study involving humans was carried out in accordance with the Declaration of Helsinki and approved by the Regional Research Ethics Committee in Qassim region (reference no. 21-18-12). Written informed consent was obtained from all participants involved in the study.

## Author contributions

MA-J: Methodology, Validation, Writing – original draft, Writing – review & editing, Conceptualization, Formal analysis, Supervision. RK: Methodology, Validation, Writing – original draft, Writing – review & editing. YA-M: Investigation, Methodology, Writing – original draft. OA-M: Investigation, Methodology, Writing – original draft. AA-S: Investigation, Writing – original draft. OA-s: Investigation, Writing – original draft. AA-s: Investigation, Writing – original draft. IA-d: Investigation, Writing – original draft. WA-E: Investigation, Writing – original draft.
